# Sustained functional composition of pollinators in restored pastures despite slow functional restoration of plants

**DOI:** 10.1002/ece3.2924

**Published:** 2017-04-19

**Authors:** Marie Winsa, Erik Öckinger, Riccardo Bommarco, Regina Lindborg, Stuart P. M. Roberts, Johanna Wärnsberg, Ignasi Bartomeus

**Affiliations:** ^1^ Department of EcologySwedish University of Agricultural SciencesUppsalaSweden; ^2^ Department of Physical GeographyStockholm UniversityStockholmSweden; ^3^ School of Agriculture, Policy and DevelopmentCentre for Agri‐Environmental ResearchUniversity of ReadingReadingUK; ^4^ Dpto. Ecología IntegrativaEstación Biológica de Doñana (EBD‐CSIC)Isla de la CartujaSevillaSpain

**Keywords:** abandonment, bees, functional diversity, habitat fragmentation, habitat restoration, hoverflies, semi‐natural grassland, trait composition

## Abstract

Habitat restoration is a key measure to counteract negative impacts on biodiversity from habitat loss and fragmentation. To assess success in restoring not only biodiversity, but also functionality of communities, we should take into account the re‐assembly of species trait composition across taxa. Attaining such functional restoration would depend on the landscape context, vegetation structure, and time since restoration. We assessed how trait composition of plant and pollinator (bee and hoverfly) communities differ between abandoned, restored (formerly abandoned) or continuously grazed (intact) semi‐natural pastures. In restored pastures, we also explored trait composition in relation to landscape context, vegetation structure, and pasture management history. Abandoned pastures differed from intact and restored pastures in trait composition of plant communities, and as expected, had lower abundances of species with traits associated with grazing adaptations. Further, plant trait composition in restored pastures became increasingly similar to that in intact pastures with increasing time since restoration. On the contrary, the trait composition of pollinator communities in both abandoned and restored pastures remained similar to intact pastures. The trait composition for both bees and hoverflies was influenced by flower abundance and, for bees, by connectivity to other intact grasslands in the landscape. The divergent responses across organism groups appeared to be mainly related to the limited dispersal ability and long individual life span in plants, the high mobility of pollinators, and the dependency of semi‐natural habitat for bees. Our results, encompassing restoration effects on trait composition for multiple taxa along a gradient in both time (time since restoration) and space (connectivity), reveal how interacting communities of plants and pollinators are shaped by different trait–environmental relationships. Complete functional restoration of pastures needs for more detailed assessments of both plants dispersal in time and of resources available within pollinator dispersal range.

## 1 Introduction

Habitat restoration aims to counteract the negative effects of habitat loss and land‐use change on biodiversity (Bakker & Berendse, 1999). Species richness and abundance have commonly been used as indicators to evaluate restoration success (Wortley, Hero, & Howes, 2013). However, while recovering community diversity in a restored habitat partly reflects a successful restoration, it does not necessarily insure that ecosystem functions such as pollination or primary production are restored (SER, 2004). To advance current research on biodiversity and ecosystem functioning, an analysis of abundance‐based trait composition of communities is a promising approach (Gagic et al., 2015). Species traits determine both how species are affected by environmental change (i.e., response traits; Lindborg et al., 2012; Öckinger et al., 2010; Williams et al., 2010) and species functional role in the ecosystem (i.e., effect traits; Lavorel & Garnier, 2002; Violle et al., 2007). In particular plant ecologists have a long tradition in attempting to generalize effects of land‐use change on communities based on species traits (Díaz et al., 2007; Lavorel, McIntyre, Landsberg, & Forbes, 1997), but also insect ecologists are increasingly applying a trait‐based approach (Moretti et al., 2017). The use of a trait‐based methods is a promising way forward also for restoration ecology (Montoya, Rogers, & Memmott, 2012). The approach has potential to disentangle how species with certain sets of traits are affected by the changes in the environment following habitat degradation and habitat restoration. This can improve our understanding of mechanisms for community assembly following habitat restoration (Helsen, Hermy, & Honnay, 2012; Laughlin, Strahan, Huffman, & Sánchez Meador, 2016).

Temperate semi‐natural grasslands harbor species‐rich plant communities (Wilson, Peet, Dengler, & Pärtel, 2012) and act as source habitat for many pollinator species (Öckinger & Smith, 2007). This habitat type has declined drastically, both globally and in many parts of Europe during the 20th century (e.g., Hoekstra, Boucher, Ricketts, & Roberts, 2005). These grasslands are therefore now highly prioritized objects for restoration (Keenleyside, Beaufoy, Tucker, & Jones, 2014). While evaluations of biodiversity restorations are increasing (Wortley et al., 2013), they often target only the plant community (e.g., McApline et al., 2016). About 87% of the world's wild flowering plant species are at least partly dependent on pollinators for their reproduction (Ollerton, Winfree, & Tarrant, 2011). Hence, to restore a functional plant community, it is vital to also consider pollinators in habitat restorations (Dixon, 2009). There are, however, surprisingly few studies on effects of restoration on key pollinator groups such as bees and hoverflies (Winfree, Bartomeus, & Cariveau, 2011). Further, while assessments of the relationship between land‐use change and functional diversity have become common (e.g., Rader, Bartomeus, Tylianakis, & Laliberté, 2014; Tscharntke et al., 2008), there is much to learn about the effect of habitat fragmentation on the functional recovery of both plants and pollinators in restored habitats. To improve our understanding of community re‐assembly in restored habitats, there is a need for simultaneous assessments of restoration effects for several taxa, and in particular, for interacting species groups (Keddy, 1992).

Community (re‐) assembly is determined by multiple species traits. One key trait determining a species’ likelihood to recolonize a restored habitat is dispersal ability. Even if all former abiotic conditions are recreated after restoration, a full recovery of community composition might be slow (Helsen, Hermy, & Honnay, 2013) or even impossible if not all species are able to reach the restored habitat (Ozinga, Bekker, Schaminee, & Van Groenendael, 2004). Therefore, when habitats in fragmented landscapes are restored, the dispersal ability of both plants and pollinators is critical for the (re)colonization and recovery of functional plant‐pollinator communities (Tscharntke & Brandl, 2004), and we predict that species with good dispersal abilities would be more likely to recolonize rapidly after restoration. Alternatively, some species might survive habitat degradation and persist as remnant populations (Hylander & Ehrlen 2013). The degree of specialization among pollinators (Bommarco et al., 2010; Williams et al., 2010) and the individual life span and seed bank longevity of plants (Lindborg, Cousins, & Eriksson, 2005), that is, “dispersal in time” (Plue & Cousins, 2013) are important traits affecting the persistence during habitat degradation, and the dynamics of populations after restoration, that is, generalist species and species with long life spans are more likely to persist in degraded habitats. Also vegetatively reproducing species might be better at persisting in degraded habitats if fecundity is reduced (Klimesova, Latzel, de Bello, & van Groenendael, 2008). Once species are present in a restored habitat, the next step is to spread within the established community, and if grasslands are grazed, to cope with grazing. Tall‐growing plants are more competitive in the absence of grazing and are expected to dominate in abandoned pastures, but plants with adaptations to grazing, for example, growing in a rosette form are predicted to benefit from restoration and re‐introduced grazing. Insects with specialized feeding behavior and nesting above ground are likely to benefit from grazing and will respond to the recovery of the plant community after restoration (Williams et al., 2010). Pollinating insects will also respond to the changed dominance patterns in herbs *vs*. grasses or woody plants and to shifts in flower traits such as flowering phenology and flower morphology. As species traits can covary or trade off in relation to one another, it is also essential to consider the relationships among traits.

**Figure 1:**
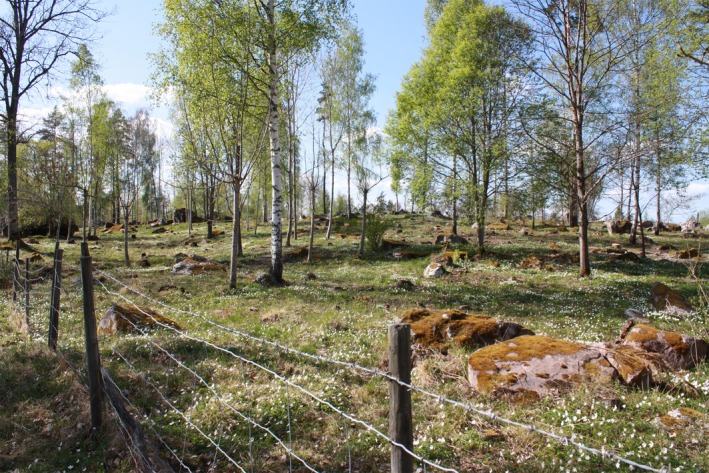
A formerly abandoned and overgrown semi‐natural pasture, restored 4 years prior to our study

Here, we use a trait‐based approach including several taxa to evaluate the restoration of semi‐natural pastures. In a landscape experiment with 38 semi‐natural pastures of different management status and history, situated along a landscape gradient, we explored how the trait composition of vascular plants and two groups of pollinating insects, hoverflies and bees, related to landscape composition, vegetation structure, and time since restoration. Specifically, we tested the hypothesis that plant and pollinator trait composition differ between intact (continuously grazed), abandoned, and restored pastures. We hypothesized that the trait composition in restored pastures is intermediate between abandoned and intact pastures, but become more similar to intact pastures with increasing time since restoration, and that this recovery is facilitated by increasing connectivity to other intact semi‐natural grasslands (Figure 1).

## 2 Methods

### 2.1 Study area

**Figure 2:**
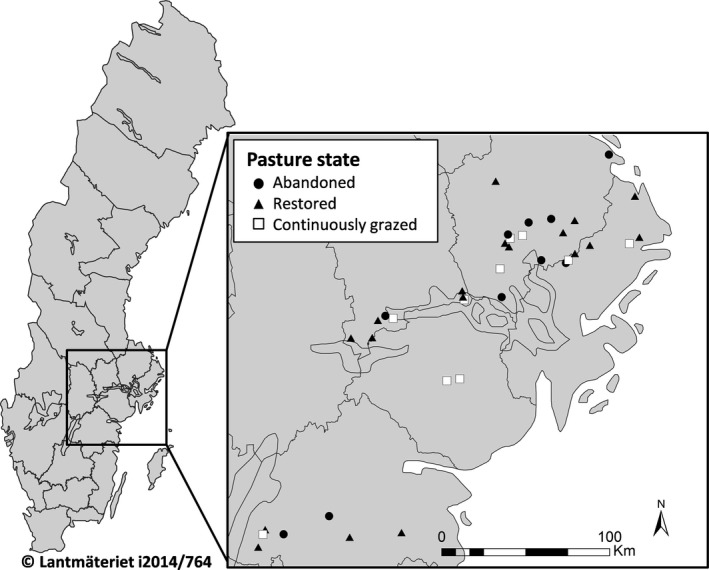
Overview map of south‐central Sweden and the location of study sites, illustrating the spatial distribution of the three pasture states included in the study design: abandoned, restored, and continuously grazed, representing a space‐for‐time substitution

To compare community composition in abandoned, restored, and intact semi‐natural pastures, we applied a space‐for‐time landscape experimental design consisting of 10 abandoned, 18 restored (formerly abandoned), and 10 continuously grazed (intact) semi‐natural pastures of dry or mesic type. The reason for the higher number of restored sites was to allow for variation in the time since restoration, so that we could specifically test for the effect of this factor. Pastures were selected along a gradient of connectivity to intact pastures in the surrounding landscapes. The pastures were situated in south‐central Sweden in the counties of Uppsala, Stockholm, Västmanland, Södermanland, and Östergötland (Figure 2). Semi‐natural pastures were defined as grasslands that depend on livestock grazing for their persistence, but with no visible signs of plowing or input of fertilizers (Eriksson, Cousins, & Bruun, 2002). These grasslands have traditionally been managed for haymaking and extensive grazing. The abandoned pastures represent the state before restoration, and the continuously grazed pastures represent the target communities which restoration actions aim at reaching. Restoration consists of clearing of shrubs and trees that during abandonment has encroached the formerly open pastures. After clearing, an extensive grazing regime is reinstated (Lindborg & Eriksson, 2004). The continuously grazed and restored pastures included in our study were grazed by cattle, horses, and/or sheep.

Abandoned and continuously grazed pastures were identified using TUVA, a national Swedish geographical database of semi‐natural grasslands (http://www.jordbruksverket.se/tuva). To standardize vegetation types between pastures as far as possible, the continuously grazed pastures were selected among habitat types classified as “Semi‐natural dry grasslands and scrublands facies on calcareous substrates,” “Fennoscandian lowland species‐rich dry to mesic grasslands,” “Lowland hay meadows,” “Fennoscandian wooded meadows,” and “Fennoscandian wooded pastures” (European Commission 2013). By restricting our selection to these habitat types, we controlled for soil conditions and land‐use history.

See more information in grassland selection in Appendix S1.

### 2.2 Survey of plants and insects

Plant communities were surveyed from June to September 2011. In each pasture, 10 randomly selected plots of 1 × 1 m were investigated in detail. As the understory vegetation was our primary focus, plots randomly assigned to dense shrubbery were moved just outside the shrubs. Each 1 m^2^ plot was divided into 100 small squares of 10 × 10 cm which were used to estimate the occurrence frequency of each vascular plant species.

Bees and hoverflies were surveyed June to August in 2011 with four visits per pasture. The pollinators were collected with sweep nets along four 50 m transects (i.e., total transect length = 200 m) per pasture and visit. Transects were permanent between visits and placed to include the habitat heterogeneity within sites. Sampling was performed during standardized weather conditions with temperatures ≥16°C, no precipitation and low wind. The time spent on each site and transect varied due to varying pollinator densities and handling time, but was fixed when handling time was discounted. Honey bees (*Apis mellifera* L.) were not abundant and were excluded from the analyses as they are managed and their density reflect hive locations rather than restoration management or landscape proprieties.

### 2.3 Landscape and habitat characteristics of pastures

For each pasture, we calculated connectivity using the index described by Hanski, Alho, and Moilanen (2000):

$${\text{CI}}_{i}=\sum \text{exp}\left(-\alpha d_{ij}\right){A_{j}}^{\beta }$$

*A*_*j*_ is the area of the neighboring fragment *j* (in hectares) and *d*_*ij*_ is the distance (in km, center to center) from the focal fragment *i* to the neighboring fragment *j*. α is a species‐specific parameter describing a species’ dispersal ability, and β describes the scaling of emigration as a function of patch area. We accounted for the distance to, and area of all other dry and mesic semi‐natural grasslands within a 5 km radius. As we applied the connectivity index to an entire community with several taxa, we set the parameter α to 1 (corresponding to an average dispersal distance of 1 km) and β to 0.5. Beta values close to 0.5 has been observed for several insect species. The rank order of pasture connectivity is not sensitive to the value of these parameters (Moilanen & Nieminen, 2002).

The area of the focal pastures is ranged from 1 to 13 ha and the connectivity from 0.1 to 26.6, with similar gradients across the pasture categories. We selected restored pastures where the restoration had taken place 1–15 years ago, with an even distribution along this time gradient (Appendix S2). For restored pastures, in addition to “pasture area,” “connectivity,” and “time since restoration,” we also estimated the proportion of tree and shrub cover in each restored pasture from aerial photographs from 2010 (Wärnsberg, 2013).

Time of abandonment was estimated by examining aerial photographs of each site with approximately 15 year intervals (first photo from the period 1959–1965; second photo 1977–1984; third photo 1992–1998; fourth photo 2011–2012). If the proportional tree and shrub cover had increased with 50% between two subsequent time periods, this was considered the period of abandonment (Wärnsberg, 2013). In cases where the tree and shrub cover already in the first aerial photo were at least 50% higher than after the restoration, we assumed that the pasture had been abandoned already before the year of the photograph, but to be conservative we assigned the year the photo was taken as the year of abandonment. The average time of abandonment before restoration was 38.9 years (*SE* = 2.0, median = 41.5; Appendix S2). Mean vegetation height in the pastures over the season and mean flower abundance (number of solitary flowers or inflorescences per plot) were calculated from three 1 × 1 m plots per transect used for the pollinators surveys (i.e., 12 plots per pasture and visit). We also validate the expectation that the three pasture states (abandoned, restored, and continuously grazed) differed in tree and shrub cover (ANOVA: *F*_2,35_ = 4.37, *p* = .020, Appendix S2, Figure a) and mean vegetation height over the season (*F*_2,35_ = 13.27, *p* = <.001, Appendix S3, Figure b), with more trees and shrubs and taller vegetation in abandoned than in restored and continuously grazed pastures. There was no difference in average flower abundance among management states (*F*_2,35_ = 1.51, *p* = .24, Appendix S3, Figure c).

### 2.4 Species traits

We examined plant and insect traits related to dispersal, reproduction, resource use and competitive ability, phenology, and (for plants) life form (Table 1), which were hypothesized to either influence species’ responses to altered habitat management, landscape connectivity, or linking the plant and pollinator communities (Table 1). For a detailed description of the traits, see Appendix S4. Bee traits were extracted from a database held by the University of Reading (primary sources for this database are listed in Appendix S4). Hoverfly traits were assembled from Syrph The Net database (Speight, Monteil, Castella, & Sarthou, 2013) and from Bartsch, Binkiewicz, Klintbjer, Råden, and Nasibov (2009a, 2009b). Plant traits were drawn from BiolFlor database (Klotz, Kühn, & Durka, 2002), from floras (Krok, Almquist, Jonsell, & Jonsell, 2013; Mossberg & Stenberg, 2003), and from our own measurements.

**Table 1:** Summary of categorical (i) and continuous trait variables (ii) included in the CWM analyses for plants, hoverflies and bees, divided into six trait types, and the rationale for including them in our analyses. For categorical variables, the number of trait levels is given in superscript. “Pollen vector” was included in analyses of both the entire plant community and of flowering forbs and shrubs, while “Color,” “Start flowering,” and “Period flowering” were included only in analyses for flowering forbs and shrubs. The traits and trait levels are further described in Appendix S4

Trait type	Rationale	Plants	Hoverflies	Bees
Dispersal	Influence recolonization ability and the relationship between connectivity and occurrence or recolonization	Diaspore type^i) 5^Pollen vector^i) 3^	Body length^ii)^ Migratory^i) 3^	Intertegular distance (ITD)^ii)^
Reproduction	Influence species persistence in abandoned pastures and recovery after restoration	Reproduction type^i) 4^ Life span^i) 4^		Sociality^i) 5^
Habitat specialist	Indicate sensitivity to altered management	Indicator species^i) 2^		
Resource use/Competition	Influence species persistence in abandoned pastures and recovery after restoration	Plant height^ii)^	Larval food^i) 3^ Adult food^i) 2^ Saproxylic^i) 2^	Lecty^i) 2^ Tongue length^i) 3^ Nesting trait^i) 4^
Life form	Influence species persistence in abandoned pastures and relationship to pollinator species	Plant type^i) 4^ Color^i) 5^ Growth form^i) 3^		
Phenology	Influence species persistence in abandoned pastures and relationship to pollinator species	Start flowering^i)^ Period flowering^ii)^	Flight start^ii)^ Flight period^ii)^	Flight start^ii)^ Flight period^ii)^

### 2.5 Statistical analyses

#### 2.5.1 Trait composition in relation to pasture state

We first characterized the trait composition in each site by calculating community‐weighted trait means (CWM) (Garnier et al., 2004) for each of the three species groups: plants, bees, and hoverflies. Analyses of plant species included the understory vegetation, while trees, woody plants, and shrubs with a maximum height of >1 m were excluded from the analyses, as these were not our primary focus and rarely are for grassland restoration purposes either. The understory vegetation, hereafter referred to as “entire plant community” was classified into grasses, forbs, sedges, and dwarf shrubs with 1 m maximum height (Mossberg & Stenberg, 2003). For categorical traits, CWM was calculated as % of individuals within the dominant trait category, except when there was a clear target category (e.g., the proportion of indicator species). Included in the CWM analyses for the entire plant community were also the proportional tree and shrub cover and mean seasonal vegetation height in the pastures. As a subset of the entire plant community dataset, flowering forbs and flowering dwarf shrubs were also analyzed separately to explore the connection between the plant community composition and composition of pollinating insects (see Appendix S5 for overview). CMW were calculated using the “FD” package (Laliberté & Shipley, 2011).

Next, to asses whether species trait composition differed among pasture states (abandoned, restored, and continuously grazed), a distance matrix among sites with pairwise comparisons of community trait composition based on euclidean distance was created using the “vegan” package (Oksanen et al., 2013). Possible differences in mean trait composition among pasture states were analyzed separately for the entire plant community, flowering plants, bees, and hoverflies using permutational analysis of variance (PERMANOVA, Anderson, 2001), and possible differences in dispersion in trait space were analyzed using permutational analysis of dispersion (PERMDISP, Anderson, 2006). To visualize trait space for the three pasture states, we used nonparametric multidimensional scaling (NMDS). PERMANOVA, PERMDISP, and NMDS were conducted using the package “vegan” (Oksanen et al., 2013).

#### 2.5.2 Species traits association to landscape and local habitat characteristics in restored pastures

Associations between species traits and landscape and local habitat variables of restored pastures were analyzed with fourth corner analysis in combination with RLQ analysis (Dray et al., 2014) using the ade4 package (Dray & Dufour, 2007). The combination of the two methods encompasses global analyses of associations between all combined environmental variables and all species traits, but specific traits that are associated to certain environmental conditions can be visually explored in the plots. This facilitates the interpretation of changes in community trait composition related to alterations in environmental factors (Dray et al., 2014).

The three ordination tables used in both RLQ and fourth corner analyses are: R (landscape and local habitat characteristics per site), L (species abundance per site), and Q (species traits; Dolédec, Chessel, ter Braak, & Champely, 1996; Legendre, Galzin, & Harmelinvivien, 1997). Landscape and local habitat variables (columns of table R) included in the analyses were “pasture area,” “connectivity,” “time since restoration” (number of years since restoration), and “abandonment time” (number of years between estimated year of abandonment and year of restoration). In the pollinator analyses, we also included “shrub cover,” “mean vegetation height” (seasonal), and “flower abundance.” These variables characterize both the physical structure of the habitat and the availability of resources for foraging and reproduction (Table 1). In the fourth corner analyses, we performed 9999 permutations using permutation model 6, which combines the permute values for site (rows of table L or R) and species abundance (columns of table L or rows of Q), and fixes the level of type I error (Dray et al., 2014). The permutation of sites test that species with fixed traits are not affected by environmental variables, while the permutation of species abundance test that fixed environmental conditions does not affect the community trait composition. No adjustments for *p*‐values were made, but due to the high number of test performed in the analyses, the possibility of significance just by chance increases. Therefore, we mainly highlight results with significance level ≤.01, and focus on the discussion of effect sizes. All statistical analyses were performed in R software, version 3.0.3 (R Development Core Team 2014).

## 3 Results

In total, 232 understory plant species were recorded in the surveys, of which 171 species were flowering forbs or shrubs. In the surveys of pollinators, a total of 870 individuals of bees and hoverflies were collected in the 38 pastures. Excluding honey bees, we recorded 55 species of bees and 54 species of hoverflies.

### 3.1 Species trait composition and trait dispersion in relation to pasture state

**Figure 3:**
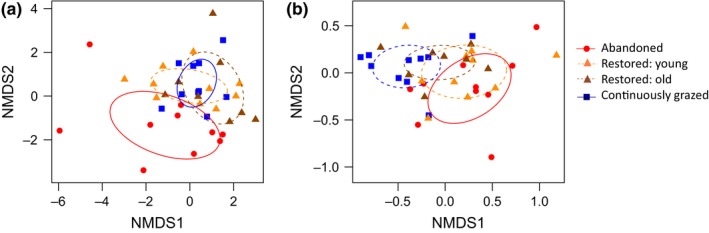
NMDS visualizing trait composition and dispersion in trait space per community, based on distance matrices of community‐weighted means (CMW) for dominating (or most relevant) trait levels, for (a) the entire plant community and (b) flowering forbs and shrubs for the tree pasture states: abandoned, restored, and continuously grazed. To visualize the effect of time since restoration, restored pastures are plotted in two age classes: “young” (restored 1–5 years prior to the study) and “old” (restored 8–15 years prior to the study). NMDS stress = 0.18 and 0.03, respectively

The trait composition of the entire plant community (PERMANOVA, *R* ^2^ = .13, *p* = .001; Figure 3a) as well as for the subset of flowering forbs and shrubs (*R* ^2^ = .24, *p* = .002; Figure 3b) differed among pasture states. For the entire plant community, the trait composition in restored pastures was similar to the trait composition in continuously grazed pastures, while the trait composition in abandoned grasslands was clearly differentiated from the other two pasture states (Figure 3a). For flowering plants, the trait composition in restored pastures took an intermediate position between abandoned and continuously grazed pastures (Figure 3b). For restored pastures, the trait composition of the entire plant community does not change with “time since restoration” (*R* ^2^ = .05, *p* = .51; Figure 3a), but there is a nonsignificant trend for flowering plants in restored pastures to become more similar to that in continuously grazed pastures with time (*R* ^2^ = .14, *p* = .10; Figure 3b).

Also the dispersion within the trait space of the entire plant community tended to differ among pasture states, that is, beta diversity in trait composition (PERMDISP, *F*_2,35_ = 2.61, *p* = .09), with the largest variability in trait composition among abandoned pastures and the smallest variability among continuously grazed pastures (Figure 3a). No such difference was found when analyzing the flowering plants only (*F*_2,35_ = 0.91, *p* = .41, Figure 3b).

**Figure 4:**
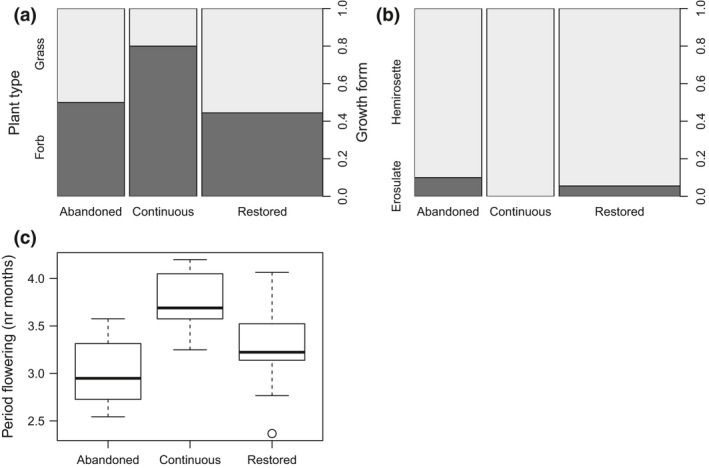
Descriptive community‐weighted means for dominant trait levels per pasture state, displaying (a) a higher proportion of domination of forbs and (b) plants with hemirosette growth form, and (c) a longer flowering period in continuously grazed pastures than in abandoned and restored pastures. The wider bar/box for restored pastures is due to larger sample size

Plant communities in continuously grazed pastures were more dominated by forbs, whereas grasses were relatively more dominating in both abandoned and restored pastures (Figure 4a). Further, in continuously grazed pastures, hemirosette plants were the sole dominant growth form, whereas in abandoned and restored pastures, the dominance in growth form also included erosulate plants (Figure 4b). The community of flowering plants in continuously grazed pastures consisted of species with an on average longer flowering period compared to abandoned and restored pastures (Figure 4c). There was no difference in average trait composition (PERMANOVA, hoverflies: *R* ^2^ = .032, *p* = .65; bees *R* ^2^ = .08, *p* = .22) or dispersion in trait composition (PERMDISP, hoverflies: *F*_2,35_ = 0.16, *p* = .85; bees: *F*_2,35_ = 0.23, *p* = .80) in the pollinator communities.

### 3.2 Species traits association to landscape and habitat characteristics in restored pastures

**Figure 5:**
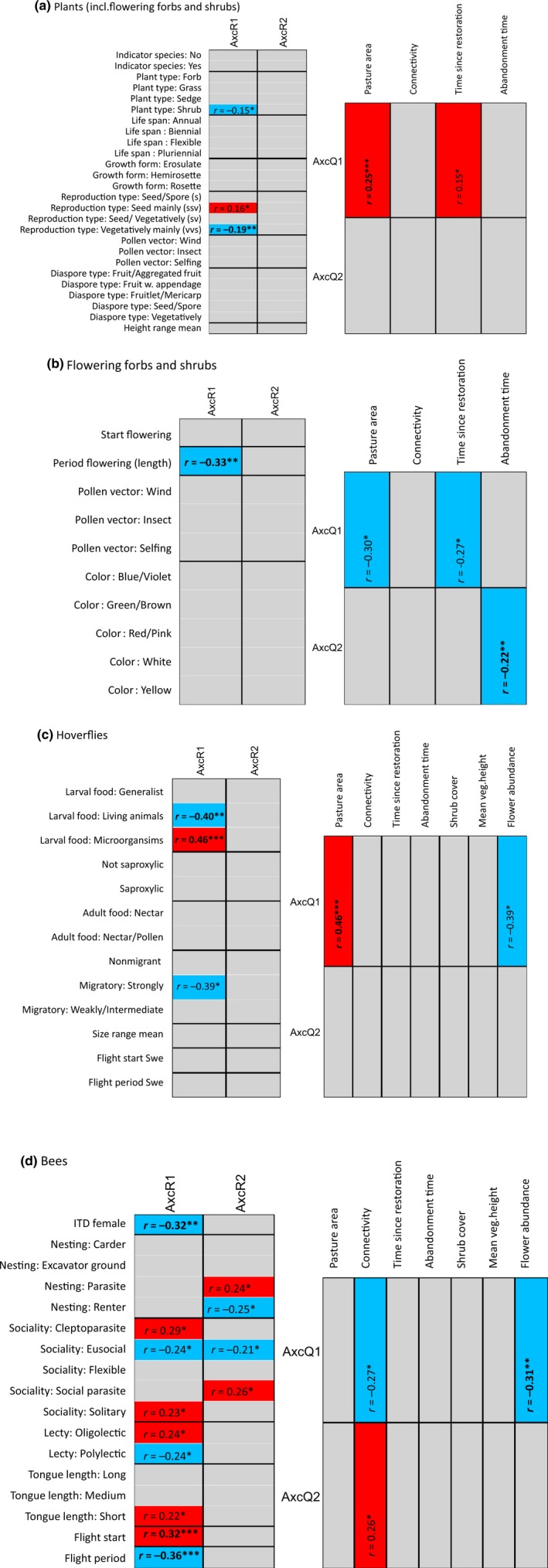
Results from the combined fourth corner and RLQ analyses for (a) the entire plant community (including flowering forbs and dwarf shrubs), (b) flowering forbs and shrubs, (c) hoverflies, and (d) bees, with effect size (*r*) and significance level (***.001, **.01, *.05). AxcR1 and AxcR2 summarize the environmental/management variables, AxcQ1 and AxcQ2 summarize species traits. Fourth corner statistics are used to test for associations between RLQ axes for environmental/management variables (AxcR1 and AxcR2) and species traits (left panel in figures), and for associations between RLQ axes for species traits (AxcQ1 and AxcQ2) and environmental/management variables (right panel in figures). Red boxes indicate positive significant associations, blue boxes negative significant associations. Associations with *p* ≤ .01 (*r* values in bold) are considered main effects

For the entire plant community (Figure 5a), the first environmental axis in the RLQ analysis (Appendix S6, Figure 1) explained 67.9% of the total variation and was mainly defined by pasture area (*p* = .001, *r* = .25) and to some degree also by time since restoration (*p* = .018, *r* = .15). This axis was significantly negatively associated with vegetatively reproducing species (*p* = .008, *r* = −.19) and was also to a lesser extent negatively associated with woody species (*p* = .020, *r* = −.15) and positively associated with species reproducing by seed (*p* = .040, *r* = .16). No traits were significantly associated with the second environmental axis (Appendix S6, Figure 1), which explained 19.3% of the variation and was weakly defined by abandonment time (*p* = .052, *r* = −.11).

For flowering plants (Figure 5b), the first environmental axis in the RLQ analysis (Appendix S6, Figure 2) explained 65.9% of the variation and was mainly defined by pasture area (*p* = .013, *r* = −.30) and to some extent by time since restoration (*p* = .036, *r* = −.27). The first axis was negatively associated with the flowering period (*p* < .001, *r* = −.33). The second environmental axis (Appendix S6, Figure 2), explaining 21.4% of the variation was defined by abandonment time (*p* = .009, *r* = −.22), but no significant associations between abandonment time and traits of flowering plants were found.

For hoverflies (Figure 5c), the first environmental axis in the RLQ analysis for hoverflies (Appendix S6, Figure 3) explained 60.9% of the variation and was defined by pasture area (*p* < .01, *r* = .46) and flower abundance (*p* = .02, *r* = −.38). This axis was positively associated with microorganism feeding larvae (*p* = <.001, *r* = .46) and negatively associated with species with predatory larvae (*p* = .002, *r* = −.40). Weaker associations to the first axis were found for strongly migratory behavior (*p* = .029, *r* = −.39) and weak to intermediately migratory behavior (*p* = .053, *r* = .39). The second environmental axis (Appendix S6, Figure 3), explaining 21.7% of the variation, was weakly defined by grassland connectivity (*p* = .054, *r* = −.27), but no significant associations between hoverfly traits and connectivity were found.

For bee communities (Figure 5d), the environmental variables defining the first axis in the RLQ analysis (Appendix S6, Figure 4), which explained 63.5% of the variation, were mainly flower abundance (*p* = .0051, *r* = −.31) and to some extent connectivity (*p* = .021, *r* = −.27). This axis was negatively associated with body size (ITD; *p* = .004, *r* = −.32) and the length of flight period (*p* < .001, *r* = −.36) but positively associated with early start of the flight period (*p* < .001, *r* = .32). Also solitary (*p* = .020, *r* = .23), eusocial (*p* = .016, *r* = −.24), cleptoparastic (*p* = .037, *r* = .29), and oligolectic behavior (*p* = .046, *r* = .24) as well as short tongue length (*p* = .024, *r* = .22) were less strongly associated to the first axis. The second environmental axis (Appendix S6, Figure 4), explaining 22.2% of the variation, was mainly defined by connectivity (*p* = .030, *r* = .26). No traits showed an association to this axis with *p* ≤ .01, but moderate association was found for nest renting species (nesting trait, *p* = .025, *r* = −.25), eusocial behavior (*p* = .046, *r* = −.21), parasitic behavior (nesting trait, *p* = .042, *r* = .24), and socially parasitic species (sociality, *p* = .027, *r* = .26). Eusocial bees show a complex pattern, with a moderate negative association to axis 1, corresponding to decreasing connectivity and flower abundance, and a weak negative association to axis 2, corresponding to increasing connectivity.

## 4 Discussion

Plant trait composition in restored semi‐natural pastures was more similar to the composition in continuously grazed (intact) pastures than that in abandoned sites, indicating that plant communities in formerly abandoned semi‐natural pastures have a good chance to recover in terms of functionality after restoration. Part of the variation in plant trait composition in restored sites, at least for flowering plants, was explained by the time since restoration, with older restored pastures being more similar to continuous pastures than recently restored pastures. In contrast to plants, the trait composition of pollinator communities was resilient to abandonment. We found no difference among pasture states for neither bee nor hoverfly trait composition. Instead, local conditions in terms of flower abundance and landscape context in terms of connectivity explained pollinator trait composition in restored pastures.

Plant communities in abandoned pastures were not only different from those in restored and intact pastures, but had also a slightly wider dispersion in trait composition. Depending on abiotic conditions such as nutrient availability, plant communities often develop in different directions after abandonment and ceased management (Bohner, Starlinger, & Koutecky, 2012). When pastures are restored, plant communities in previously abandoned pastures gradually evolve toward a trait composition more similar to that in intact pastures, which are characterized by a high proportion of species with grazing adaptations (Bullock et al., 2001; Díaz et al., 2007), that is, forbs dominating over grasses and plants with a hemirosett growth form dominating over other growth forms. However, the process of plant community recovery following restoration is a slow process (Helsen et al., 2013), and within the time frame of the current study, the dispersion in trait space was still wider among restored pastures than in the target community. Old‐restored pastures were indeed closer to intact pastures in flowering plant composition than recently restored ones, but an even longer perspective is needed to clarify whether all restored pastures would be able to reach the conditions of the intact pastures.

We found no effect of connectivity to intact grasslands on plant trait composition. However, grassland specialist plant species often have limited ability to disperse over large distances (Verkaar, Schenkeveld, & Klashorst, 1983), and source habitats for plant community recovery in restored pastures are restricted to adjacent intact grasslands (Winsa, Bommarco, Lindborg, Marini, & Öckinger, 2015). Therefore, the main mechanisms for plant community recovery after restoration are more likely to be gradual spread from remnant populations surviving abandonment (Lindborg & Eriksson, 2004) or recolonization from the seed bank (Fagan, Pywell, Bullock, & Marrs, 2008). Concordantly, the positive effect of habitat area on seed dispersing species could be a result of an increased number of persisting individuals during abandonment before restoration (Harrison & Bruna, 1999; but see Lindborg et al., 2012).

The plant community provides the resource base for pollinating insects. Even though the traits included were not identical for plants and pollinators, we expected to find similar patterns in the community recovery of plants and pollinators in the restored pastures (c.f. Clough et al., 2014), but such connections were relatively weak in our study system. Despite the differences in plant trait composition between intact, abandoned, and restored pastures and the changes in plant trait composition with time since restoration, we found no such patterns in pollinator trait composition. Nevertheless, the pollinator trait composition within restored pastures was influenced by resource availability. For bees, an increased flower abundance tended to be related to a shift in average bee feeding specialization with more generalist (polylectic) compared to specialist (oligolectic) bees. The observation that restored and continuously grazed pastures were similar in flower availability helps explain why pollinator trait composition is maintained among pasture states. Abandoned and restored pastures could then still act as important foraging or nesting habitat for diverse pollinators (Dunning, Danielson, & Pulliam, 1992).

The exploration of trait–environmental relationships of pollinators in restored pastures revealed that connectivity to intact grasslands had no effect on hoverfly trait composition, but had a moderate influence on the trait composition of bees. There is piling evidence that while hoverfly abundance is not affected by isolation from species‐rich grasslands, bee abundance decreases (Ekroos, Rundlöf, & Smith, 2013; Rader et al., 2016; Steffan‐Dewenter & Tscharntke, 1999; but see Öckinger, Lindborg, Sjödin, & Bommarco, 2012 *)*. The differing response to connectivity between bees and hoverflies can be explained both by species mobility and resource use. Our results indicate that bees with small body size, reflecting low mobility (Greenleaf, Williams, Winfree, & Kremen, 2007), are absent from the most isolated of the restored pastures. In contrast, most hoverflies are relatively mobile and not structured by landscape composition. Further, while bees are central place foragers that rely on resources within reach from their nesting site, hoverflies can more freely track resources across the agricultural landscape (Ekroos et al., 2013).

Using a recent analysis method (Dray et al., 2014), where covariation in trait–environmental relationships is included, we could evaluate restoration outcomes for semi‐natural pastures along a gradient in both space (connectivity) and time (since restoration) from a functional perspective. Trait‐based approaches reveal important mechanisms that are at play in the dis‐ and re‐assembly of communities in degraded and restored habitats, which in turn can greatly contribute to successful planning and realization of habitat restorations. The contrasting trait responses to abandonment and restoration of semi‐natural pastures between plants and pollinators that we found highlight that the community composition of these taxa depends on processes occurring on different spatial and temporal scales. Plant community functional recovery is a slow process, and communities had not fully recovered 15 years after restoration. In contrast, pollinating insect communities can apparently maintain their functional diversity through abandonment and restoration if sufficient resources for survival and reproduction are available in the landscape, and if the necessary physical conditions are restored locally. To fully understand the mechanisms behind trait responses of plants and pollinators in restored habitats, there is a need for more detailed assessments of plant dispersal in time (seed bank longevity, e.g., Auffret & Cousins, 2011), and of resource use and the distribution of resources for pollinators both in degraded and restored habitats and at a landscape scale.

## Conflict of Interest

None declared.
