# Supplementary material for: Sustained functional composition of pollinators in restored pastures despite slow functional restoration of plants
Source: Ecol Evol. 2017 Apr 19;7(11):3836–46. doi: 10.1002/ece3.2924 (PMC5468136; doi:10.1002/ece3.2924)
Supplement: Supplementary file 1 [file ECE3-7-3836-s001.docx]

*Slow functional restoration of plants in semi-natural pastures, despite pollinators are sustained through landscape effects*, Ecology and Evolution.

Winsa M., Öckinger E, Bommarco R., Lindborg R., Roberts S. P. M., Wärnsberg J., Bartomeus I.

### Appendix S1. Grassland selection

Abandoned and continuously grazed pastures were identified using TUVA, a national Swedish geographical database of semi-natural grasslands (<http://www.jordbruksverket.se/tuva>). To standardize vegetation types between pastures as far as possible, the continuously grazed pastures were selected among habitat types classified as ‘Semi-natural dry grasslands and scrublands facies on calcareous substrates’, ‘Fennoscandian lowland species-rich dry to mesic grasslands’, ‘Lowland hay meadows’, ‘Fennoscandian wooded meadows’ and ‘Fennoscandian wooded pastures’ (European Commission 2013). The Uppland foundation, the County boards and a number of municipalities in the region provided us with information to locate and select restored pastures where the landowners had received economic compensation to restore abandoned pastures.

The area of the focal pastures ranged from 1 to 13 ha, with similar gradients in area across the pasture categories. The mean area for the three pasture states were 2.6 ha (SE= 0.2, median= 2.7) for abandoned pastures, 3.8 ha (SE=0.7, median= 3.3) for restored pastures and 2.8 ha (SE=0.5, median 2.3) for continuously grazed pastures. Connectivity ranged from 0.1 to 26.6. The mean connectivity was 8.7 (SE= 2.2, median= 7.3) for abandoned pastures, 8.3 (SE= 1.8, median= 5.6) for restored pastures and 7.6 (SE= 2.3, median= 6.9) for continuously grazed pastures (Supporting information, Appendix S1). The gradient in proportion of forest in the landscape (5 km radius) was similar among pasture states: abandoned 14-62%, restored 23-76% and continuously grazed 23-63% (not included in analysis).

European Commission. (2013) *Interpretation Manual of European Union Habitats*. EUR 28. DG Environment, Nature ENV B.3.
