# Supplementary material for: Sustained functional composition of pollinators in restored pastures despite slow functional restoration of plants
Source: Ecol Evol. 2017 Apr 19;7(11):3836–46. doi: 10.1002/ece3.2924 (PMC5468136; doi:10.1002/ece3.2924)
Supplement: Supplementary file 2 [file ECE3-7-3836-s002.docx]

*Slow functional restoration of plants in semi-natural pastures, despite pollinators are sustained through landscape effects*, Ecology and Evolution.

Winsa M., Öckinger E, Bommarco R., Lindborg R., Roberts S. P. M., Wärnsberg J., Bartomeus I.

**Appendix S2**. Information on area and connectivity for all pastures, and for abandoned and restored pastures on year of abandonment and time of abandonment until the surveys were performed or restoration started respectively. For restored pastures the table also includes information on year when restoration started and time passed between year of restoration until study was performed. In ‘State of pasture’ for restored sites “young” and “old” refers to sub groups used for visualisation of effect of time since restoration on trait composition. Year of abandonment was estimated from aerial photographs (Wärnsberg 2013).

| **State of pasture** | **Site** | **Area**  **(ha)** | **Connectivity** | **Abandoned (year)** | **Abandonment time (years)** | **Restored (year)** | **Time since restoration**  **(years)** |
| --- | --- | --- | --- | --- | --- | --- | --- |
| Abandoned | Eke Vidbo | 2.8 | 2.2 | 1960 | 51 |  |  |
| Abandoned | Forkarby | 2.8 | 3.2 | 1989 | 22 |  |  |
| Abandoned | Grän Rasbo | 2.7 | 16.2 | 1968 | 43 |  |  |
| Abandoned | Haknäs Vassunda | 1.8 | 3.4 | 1971 | 40 |  |  |
| Abandoned | Jordmarken Kungsör | 3.6 | 11.2 | 1960 | 51 |  |  |
| Abandoned | Kungs-Husby | 2.7 | 2.8 | 1971 | 40 |  |  |
| Abandoned | Långalma Öregrund | 2.9 | 14.0 | 1968 | 43 |  |  |
| Abandoned | Norräng Ryckelsby | 2.5 | 20.3 | 1990 | 21 |  |  |
| Abandoned | OpplundaVikingstad | 2.4 | 12.5 | 1962 | 49 |  |  |
| Abandoned | Storsätra Vänge | 1.8 | 1.5 | 1960 | 51 |  |  |
| Restored (young) | Ahlezons Hage | 5.4 | 1.3 | 1972 | 37 | 2009 | 2 |
| Restored (old) | Ändeberga Bålsta | 4.5 | 4.5 | 1971 | 30 | 2001 | 10 |
| Restored (young) | Bonderyd Ödeshög | 2.6 | 23.3 | 1962 | 46 | 2008 | 3 |
| Restored (young) | Borgardalsbadet | 1.0 | 1.6 | 1960 | 48 | 2008 | 3 |
| Restored (young) | Bullerum | 13.0 | 0.1 | 1963 | 46 | 2009 | 2 |
| Restored (old) | Focksta 2 | 7.6 | 7.4 | 1960 | 40 | 2000 | 11 |
| Restored (old) | Forsbacka Rimbo | 2.5 | 5.7 | 1987 | 14 | 2001 | 10 |
| Restored (young) | Johns Hage Valla | 4.2 | 26.6 | 1972 | 36 | 2008 | 3 |
| Restored (old) | Malmberga Häverö | 1.7 | 0.4 | 1960 | 42 | 2002 | 9 |
| Restored (young) | Reutersberg | 2.8 | 13.1 | 1960 | 47 | 2007 | 4 |
| Restored (old) | Röcksta Norrtälje | 1.1 | 3.8 | 1960 | 43 | 2003 | 8 |
| Restored (old) | Sjöängen Engsö | 5.1 | 13.8 | 1968 | 28 | 1996 | 15 |
| Restored (young) | Södra Lunger | 4.1 | 1.5 | 1960 | 46 | 2006 | 5 |
| Restored (young) | Stora Tadinge | 1.4 | 10.6 | 1968 | 42 | 2010 | 1 |
| Restored (young) | Stubbetorp | 3.8 | 11.8 | 1972 | 36 | 2008 | 3 |
| Restored (young) | Tjälinge Skogstibble | 0.8 | 2.2 | 1972 | 35 | 2007 | 4 |
| Restored (old) | Tjärns Hage | 5.2 | 15.7 | 1959 | 41 | 2000 | 11 |
| Restored (old) | Tyringe | 1.7 | 5.5 | 1960 | 43 | 2003 | 7 |
| Continuously grazed | Blänkhemshagen Kungsör | 2.5 | 7.2 |  |  |  |  |
| Continuously grazed | Ekeby Norrtälje | 0.8 | 1.2 |  |  |  |  |
| Continuously grazed | Graneberg Litslena | 2.7 | 7.7 |  |  |  |  |
| Continuously grazed | Kramnäs | 1.8 | 8.5 |  |  |  |  |
| Continuously grazed | Läby | 1.7 | 2.2 |  |  |  |  |
| Continuously grazed | Långholmsbryggan Engsö | 5.9 | 8.8 |  |  |  |  |
| Continuously grazed | Norrby Vidbo | 3.6 | 5.9 |  |  |  |  |
| Continuously grazed | Paris Vänge | 2.1 | 1.6 |  |  |  |  |
| Continuously grazed | Sättra Ög | 1.8 | 26.4 |  |  |  |  |
| Continuously grazed | Sparreholm | 5.4 | 6.6 |  |  |  |  |

Wärnsberg, J. (2013) Plant species richness in abandoned and restored grazed grasslands : effect of degree of overgrowth and time since overgrowth started. *Bachelor thesis. SLU Department of ecology*, **8**.
