# Supplementary material for: Sustained functional composition of pollinators in restored pastures despite slow functional restoration of plants
Source: Ecol Evol. 2017 Apr 19;7(11):3836–46. doi: 10.1002/ece3.2924 (PMC5468136; doi:10.1002/ece3.2924)
Supplement: Supplementary file 3 [file ECE3-7-3836-s003.docx]

*Slow functional restoration of plants in semi-natural pastures, despite pollinators are sustained through landscape effects*, Ecology and Evolution.

Winsa M., Öckinger E, Bommarco R., Lindborg R., Roberts S. P. M., Wärnsberg J., Bartomeus I.

**Appendix S3**


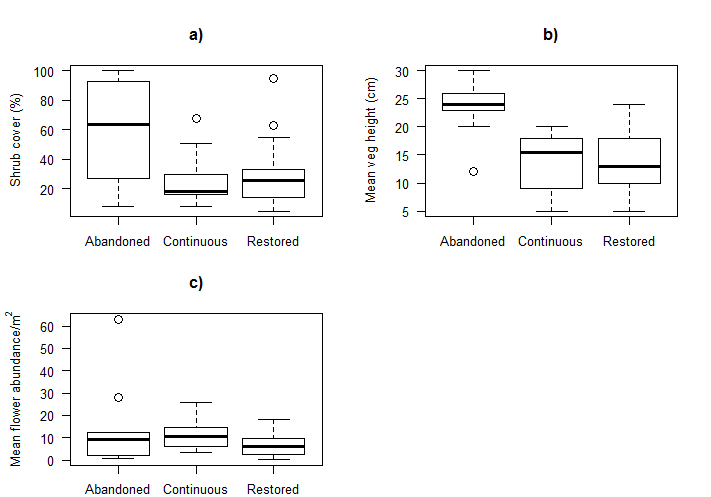


Boxplots of 1) tree and shrub cover, 2) mean vegetation height and 3) mean seasonal flower abundance per m^2^ among pasture states. The three pasture states differed in tree and shrub cover and mean vegetation height over the season, with more trees and shrubs and taller vegetation in abandoned than in restored and continuously grazed pastures (Fig. a, b). There was no difference in flower abundance among management states (Fig. c).
