# Supplementary material for: Sustained functional composition of pollinators in restored pastures despite slow functional restoration of plants
Source: Ecol Evol. 2017 Apr 19;7(11):3836–46. doi: 10.1002/ece3.2924 (PMC5468136; doi:10.1002/ece3.2924)
Supplement: Supplementary file 4 [file ECE3-7-3836-s004.docx]

*Slow functional restoration of plants in semi-natural pastures, despite pollinators are sustained through landscape effects*, Ecology and Evolution.

Winsa M., Öckinger E., Bommarco R., Lindborg R., Roberts S. P. M., Wärnsberg J., Bartomeus I.

**Appendix S4**. Species traits included in the analyses, with trait levels used and a description of each trait level.

| **Plant trait** | **Trait levels** | **Explanation** |
| --- | --- | --- |
| Diaspore type | Fruit/aggregated fruit  Fruit with appendage  Fruitlet/Mericarp  Seed/Spore  Vegetative | *Fruit/aggregated fruit:* Developed from the ovary, contains the seeds/From several fruitlets from a single flower.  *Fruit with appendage:* Fruit connected to part of plant to support dispersal or germination.  *Fruitlet/Mericarp:* Part of aggregate fruit, from single carpel, contains all fruit layers/ One segment of a fruit, breaks into units from carpels.  *Seed/Spore:* Embryo from by pollen fertilized ovule, outer coat, contains often nutrients/ Gamete that develops into new organism without fertilization.  *Vegetative:* primarily vegetative reproduction and not through fruit (Klotz *et al.* 2002) |
| Pollen vector | Insect  Wind  Selfing | *Insect*: Pollination by insects.  *Wind*: Pollination by wind.  *Selfing*: Spontaneous pollination within a flower (Klotz *et al.* 2002) |
| Reproduction type | Seed/spore (s)  Seed mainly (ssv)  Seed/Vegetatively (sv)  Vegetatively mainly (vvs) | *Seed/spore:* By seed or spore.  *Seed mainly:* Mainly by seed, rarely vegetatively.  *Seed/Vegetatively:* By seed or vegetatively  *Vegetatively mainly:* Mainly vegetatively, rarely by seed (Klotz *et al.* 2002) |
| Life span | Annual  Biennial  Pluriennial  Flexible | *Annual*: Maximum one year cycle  *Biennial*: Appr. one year of vegetative growth, thereafter generative phase an completion of cycle.  *Pluriennial*: more than one year of vegetative growth before generative phase an completion of cycle (Klotz *et al.* 2002). A plant defined in more than one level was considered “flexible”. |
| Indicator species | Yes  No | Considered grassland indicator species by the Swedish Board of Agriculture |
| Plant height | Continuous | The median value of the height range (Mossberg & Stenberg 2003) |
| Plant type | Forb  Grass  Sedge  Shrub |  |
| Colour | Blue/Violet  Green/Brown  Red/pink  White/Yellow | *Blue/Violet*: Blue, violet or purple flowers  *Green/Brown*: Green or brown flowers  *Red/pink*: Red or pink flowers  *White/Yellow*: White or yellow flowers.  Colors defined by Klotz *et al.* (2002). |
| Growth form | Erosulate  Hemirosette  Rosette | *Erosulate:* long internodes, leaves arranged equally spaced from each other on the axes.  *Hemirosette*: Change between phases of either long or short internodes, leaves either scattered or packed at the shoot. If defined in database as both hemirosette and erosulate or rosette, here defined as hemirosette.  *Rosette*: Short internodes, all foliar leaves forming a rosette, only flower stalk with long internodes (Klotz *et al.* 2002) |
| Start flowering | Continuous | Month when flowering species begin to bloom in Scandinavia (Mossberg & Stenberg 2003) |
| Period flowering | Continuous | Nr of months when flowering species are in bloom in Scandinavia. (Mossberg & Stenberg 2003) |
| **Hoverfly trait** | **Trait levels** | **Explanation** |
| Body length | Continuous | The median value of the body length range indicated by Nationalnyckeln (Bartsch *et al.* 2009a; b) |
| Migratory | Non migrant  Strongly  Weakly/Intermediate | Species categorized on basis of the extent to which they undertake long-distance movements (Speight *et al.* 2013) |
| Larval food | Generalist  Living animals  Microorgansims | *Generalist*: Species whose larvae are predatory and feed on plants or microorganisms, or species that feed on microorganisms and plants.  *Living* *animals*: Species whose larvae are predatory  *Microorgansims*: Species whose larvae feed on microorgansims associated with various forms of decomposing organic matter.  (Speight *et al.* 2013) |
| Adult food | Nectar  Nectar/Pollen | *Nectar*: Nectar only  *Nectar/Pollen*: Nectar or pollen (Speight *et al.* 2013) |
| Saproxylic | Saproxylic  Not saproxylic | *Saproxylic*: Dead wood associated species, either dependent upon dead or dying wood or upon other saproxylics (Speight *et al.* 2013) |
| Flight start | Continuous | Start of flight season(month) in Scandinavia (Bartsch *et al.* 2009a; b) |
| Flight period | Continuous | Length of flight season (nr of months) in Scandinavia (Bartsch *et al.* 2009a; b) |
| **Bee trait** | **Trait levels** | **Explanation** |
| Body size  (Inter-tegular distance, ITD) | Continuous | Distance between a females tegulae, the point where the wings are attached to thorax (Roberts S.P.M. & Potts S.) |
| Sociality | Cleptoparasite  Eusocial  Flexible  Social parasite  Solitary | *Cleptoparasite*: Uses the nest of other solitary species for reproduction  *Eusocial*: Colony living species  *Flexible*: Species with varying social traits (i.e. Eusocial in parts of range and solitary in other parts.)  *Social* *parasite*: Uses other species colonies for reproduction  *Solitary*: Female constructs own nest (Roberts S.P.M. & Potts S.) |
| Lecty | Oligolectic  Polylectic | *Oligolectic*: Uses a narrow range of plant species as pollen source (may be a single plant family or even genus)  *Polylectic*: Uses a broad range of species as pollen sources (Roberts S.P.M. & Potts S.) |
| Tongue length | Long  Medium  Short | *Long*: Long tongued bumble bees  *Medium*: Long tongued bees and short tongued bumble bees  *Short*: Short tongued bees  (Roberts S.P.M. & Potts S.) |
| Nesting trait | Carder  Excavator ground  Parasite  Renter | *Carder*: Builds brood cells from shredded plant fibre (some *Bombus* only)  *Excavator ground*: Excavates nest in the ground  *Parasite*: Uses other species nests or colonies  *Renter*: Uses existing cavities  (Roberts S.P.M & Potts S.) |
| Flight start | Continuous | Start of flight season (month) (Roberts S.P.M. & Potts S.) |
| Flight period | Continuous | Length of flight season (nr of months) (Roberts S.P.M. & Potts S.) |

**References:**

Hoverfly traits were assembled from:

Bartsch, H., Binkiewicz, E., Klintbjer, A., Råden, A. & Nasibov, E. (2009a) *Nationalnyckeln till Sveriges Flora Och Fauna. Tvåvingar: Blomflugor: Eristalinae & Microdontinae. Diptera: Syrphidae: Eristalinae & Microdontinae*. ArtDatabanken, SLU, Uppsala.

Bartsch, H., Binkiewicz, E., Klintbjer, A., Råden, A. & Nasibov, E. (2009b) *Nationalnyckeln till Sveriges Flora Och Fauna. Tvåvingar: Blomflugor: Syrphinae. Diptera: Syrphidae: Syrphinae.* ArtDatabanken, SLU, Uppsala.

Speight, M.C.D., Monteil, C., Castella, E. & Sarthou, J.-P. (2013) StN 2013. *Syrph the Net on CD, Issue 9, the database of European Syrphidae* (eds M.C.D. Speight, C. Monteil, E. Castella & J.-P. Sarthou), Syrph the Net Publications, Dublin.

Plant traits were assembled from:

Klotz, S., Kühn, I. & Durka, W. (2002) BIOLFLOR - Eine Datenbank mit biologisch-ökologischen Merkmalen zur Flora von Deutschland. *Schriftenreihe für Vegetationskunde* pp. 1–334.

Krok, T.O.B.., Almquist, S., Jonsell, L. & Jonsell, B. (2012) Svensk Flora, 29th ed (eds S Lundquist and A Rhode). Liber AB, Stockholm.

Mossberg, B. & Stenberg, L. (2003) Den Nya Nordiska Floran. Wahlström & Widstrand, Tangen.

Bee traits were provided by Stuart P.M. Roberts and Simon Potts, from a database held by University of Reading. The primary sources of information for this data base are:

Banaszak, J., Cierzniak, T., & Radchenko, V. G. E. (2000). *Bees of the family Halictidae (excluding Sphecodes) of Poland: taxonomy, ecology, bionomics*. Wydawnictwo Uczelniane Wyższej Szkoły Pedagogicznej w Bydgoszczy.

Banaszak, J., & Romasenko, L. (1998). *Megachilid bees of Europe (Hymenoptera, Apoidea, Megachilidae)*. Pedagogical University.

Dylewska, M. (1987). Die Gattung *Andrena* Fabricius (Andrenidae, Apoidea) in Nord- und Mitteleuropa. Acta zool. cracov., 30, 359–708

Falk, S. (1991). *A review of the scarce and threatened bees, wasps and ants of Great Britain* (No. 35). Nature Conservancy Council for England.

Løken, A. (1973). *Studies on Scandinavian bumble bees (Hymenoptera, Apidae).* Norsk entomologisk Tidsskrift, 20: 1–218.

Peeters, T., Raemakers. I, & Smit, J (1999) *Voorlopige atlas van de Nederlandse bijen (Apidae)*. Stichting European Invertebrate Survey, Leiden.

Söderman G., Leinonen R. (2003) *Suomen mesipistiäisetja niiden uhanalaisuus*. Helsinki

Westrich, P. (1990). *Wildbienen Baden-Württembergs*. E. Ulmer.
