# Supplementary material for: Sustained functional composition of pollinators in restored pastures despite slow functional restoration of plants
Source: Ecol Evol. 2017 Apr 19;7(11):3836–46. doi: 10.1002/ece3.2924 (PMC5468136; doi:10.1002/ece3.2924)
Supplement: Supplementary file 5 [file ECE3-7-3836-s005.docx]

*Slow functional restoration of plants in semi-natural pastures, despite pollinators are sustained through landscape effects*, Ecology and Evolution.

Winsa M., Öckinger E., Bommarco R., Lindborg R., Roberts S. P. M., Wärnsberg J., Bartomeus I.

**Appendix S5.**

Table 1. Overview of main analyses including aims of analyses, the level on which analyses were performed, and the variables included for each species group. Note that ‘Flowering plants’ is a subset of ‘Entire plant community’.

| Analysis | Aim of analysis | Level of analysis | Species groups | Variables included |
| --- | --- | --- | --- | --- |
| PERMANOVA | Difference in trait composition | Among all pasture states | 1a) Entire plant community  1b) Flowering plants  1c) Hoverflies  1d) Bees | 1a) Species traits  Shrub cover  Mean vegetation height  1b-d) Species traits |
| 4^th^ corner/  RLQ | Associations between species traits and landscape and local habitat variables | Within restored pastures | 2a) Entire plant community  2b) Flowering plants  2c) Hoverflies  2d) Bees | 2a-d) Pasture area  Connectivity  Time since restoration  Abandonment time  2c,d) All above +  Shrub cover  Mean vegetation height  Flower abundance |
