# Supplementary material for: Sustained functional composition of pollinators in restored pastures despite slow functional restoration of plants
Source: Ecol Evol. 2017 Apr 19;7(11):3836–46. doi: 10.1002/ece3.2924 (PMC5468136; doi:10.1002/ece3.2924)
Supplement: Supplementary file 6 [file ECE3-7-3836-s006.docx]

*Slow functional restoration of plants in semi-natural pastures, despite pollinators are sustained through landscape effects*, Ecology and Evolution.

Winsa M.Öckinger E., , Bommarco R., Lindborg R., Roberts S. P. M., Wärnsberg J., Bartomeus I.

**Appendix S6.**


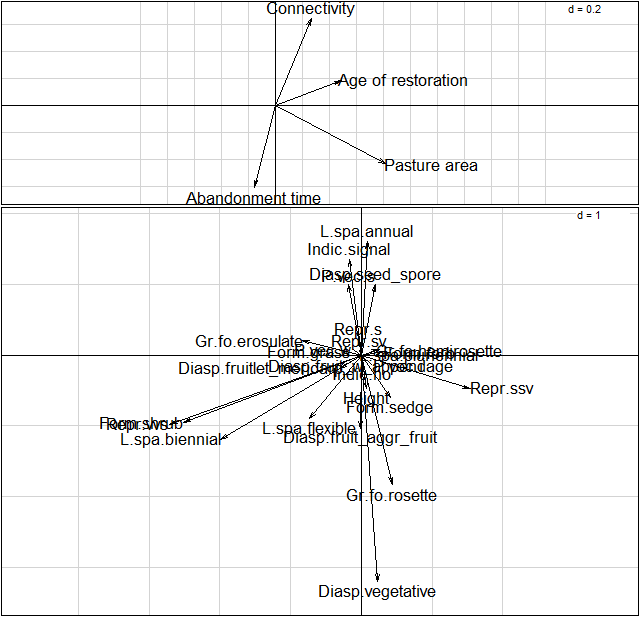


1. RLQ axes for the entire plant community. The first environmental axis was mainly defined by pasture area and to some degree also by time since restoration. This axis was significantly negatively associated with vegetatively reproducing species, and was also to a lesser extent negatively associated with woody species and positively associated with species reproducing by seed. No traits were significantly associated with the second environmental axis, which was weakly defined by abandonment time.


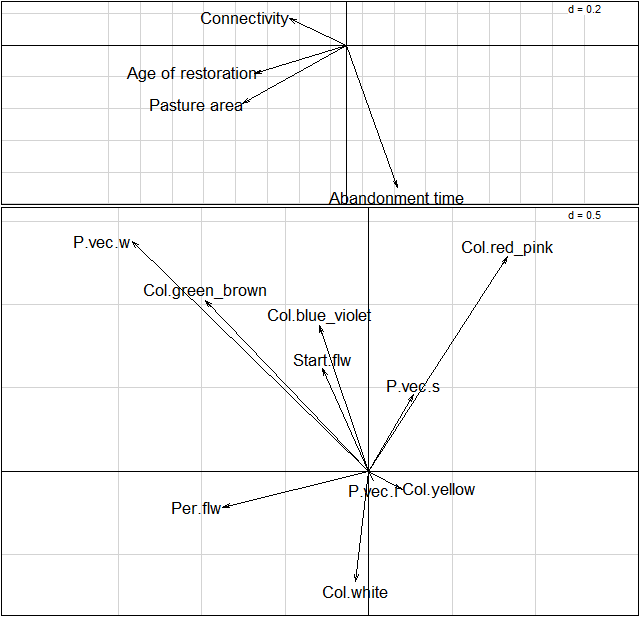


1. RLQ axes for flowering plants. The first environmental was mainly defined by pasture area, and to some extent by time since restoration. The first axis was negatively associated with the flowering period. The second environmental axis was defined by abandonment time but no significant associations with flowering traits were found.


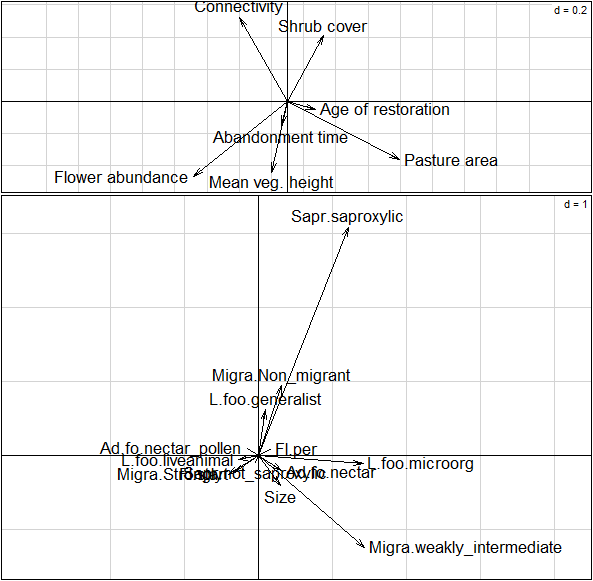


1. RLQ axes for hoverflies. The first environmental axis was defined by pasture area and flower abundance. This axis was positively associated with microorganism feeding larvae and negatively associated with species with predatory larvae. Weaker associations to the first axis were found for strongly migratory behaviour (negative) and weak to intermediately migratory behaviour (positive). The second environmental axis was defined by grassland connectivity, but no significant associations between hoverfly traits and connectivity were found.


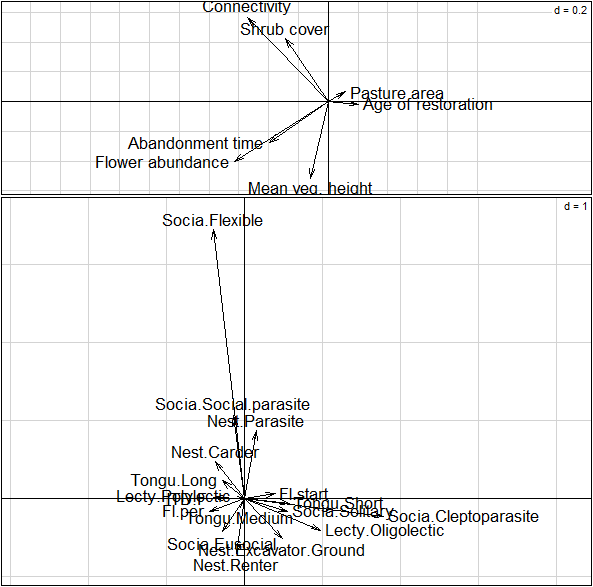


1. RLQ axes for bees. The environmental variables defining the first axis in the RLQ analysis were mainly flower abundance and to some extent connectivity. This axis was negatively associated with body size (ITD) and the length of flight period, but positively associated with early start of the flight period. Also solitary (positive), eusocial (negative), cleptoparasitic (positive) and oligolectic behaviour (positive) as well as short tongue length (positive) were less strongly associated to the first axis. The second environmental axis was mainly defined by connectivity. No traits showed an association to this axis with p≤0.01, but moderate association was found for nest renting species (negatively), parasitic behaviour (nesting trait, positively) and socially parasitic species (sociality, positively). Eusocial bees show a complex pattern, with a moderate negative correlation to axis 1, corresponding to decreasing connectivity and flower abundance, and a weak negative correlation to axis 2, corresponding to increasing connectivity.
